# Gaze Direction and Request Gesture in Social Interactions

**DOI:** 10.1371/journal.pone.0036390

**Published:** 2012-05-31

**Authors:** Alessandro Innocenti, Elisa De Stefani, Nicolò Francesco Bernardi, Giovanna Cristina Campione, Maurizio Gentilucci

**Affiliations:** 1 Department of Neuroscience, University of Parma, Parma, Italy; 2 Rete Multidisciplinare Tecnologica, Istituto Italiano di Tecnologia and University of Parma, Parma, Italy; 3 Department of Psychology, University of Milano-Bicocca, Milano, Italy; University of Bologna, Italy

## Abstract

One of the most important faculties of humans is to understand the behaviour of other conspecifics. The present study aimed at determining whether, in a social context, request gesture and gaze direction of an individual are enough to infer his/her intention to communicate, by searching for their effects on the kinematics of another individual's arm action. In four experiments participants reached, grasped and lifted a bottle filled of orange juice in presence of an empty glass. In experiment 1, the further presence of a conspecific not producing any request with a hand and gaze did not modify the kinematics of the sequence. Conversely, experiments 2 and 3 showed that the presence of a conspecific producing only a request of pouring by holding the glass with his/her right hand, or only a request of comunicating with the conspecific, by using his/her gaze, affected lifting and grasping of the sequence, respectively. Experiment 4 showed that hand gesture and eye contact simultaneously produced affected the entire sequence. The results suggest that the presence of both request gesture and direct gaze produced by an individual changes the control of a motor sequence executed by another individual. We propose that a social request activates a social affordance that interferes with the control of whatever sequence and that the gaze of the potential receiver who held the glass with her hand modulates the effectiveness of the manual gesture. This paradigm if applied to individuals affected by autism disorder can give new insight on the nature of their impairment in social interaction and communication.

## Introduction

During social interactions, a person is able to communicate nonverbally intentions and attitudes via gestures. Manual gesture is a powerful form of nonverbal communication [1] and it allows individuals to communicate a variety of feelings and thoughts. However, other sources of nonverbal communication, such as facial expressions, may intercede in order to insure successful communication during social interactions. The eyes dominate facial expressions and eye contact is used as a signal to convey willingness to interact [2], [3]. A lot of studies have revealed a network of structures involved in human social interaction and communication, named ‘the social brain” (see for example [4], [5]). The social brain is a cortical and subcortical network of regions, including ventral and medial prefrontal cortex, superior temporal gyrus, fusiform gyrus, cingulate gyrus and amygdala, which are specialized to process social information such as the face, gaze, biological motion, and human action [3]–[8]. It is possible that eye contact modulates the development and activation of the social brain network; nevertheless, the precise manner in which these areas interact to guide social behaviour remains unclear.

A particular type of gesture, the request gesture, besides conveying communicative intents, is more interactional, being used to initiate, maintain, regulate, or terminate various types of interaction. It is also called instrumental gesture, i.e. designed to influence the immediate behavior of another [9]. Recently, a not yet published fMRI study (Ferri, Busiello, Campione, Romani, Costantini, Gentilucci, unpublished results), has investigated the cortical activations when request gestures were produced with and without gaze availability. The authors found a right-lateralized network strongly activated by the observation of request gestures performed by the blindfolded as compared to the not-blindfolded actor. Similar results were observed by Pierno et al. [10]. In their study participants observed two agents performing either cooperative or individual actions. The two agents were either blindfolded or not. They found activation of right dorsal-medial-prefrontal cortex for observation of cooperative action in the condition of blindfolded agent, whereas Ferri et al. (unpublished data) found activation of right dorso-lateral-prefrontal-cortex in the same condition of blindfolded agent. These data suggest that gaze is mandatory for grabbing the social intention beyond actions performed by conspecifics. Right prefrontal cortex activation probably reflects the effort to fully understand the actors’ potential interest to interact, when relevant social cues (i.e. gaze) are missing. The finding that different areas of prefrontal cortex were activated probably depends on the type of interaction coded by the actions of actor(s). As a consequence of these results, in the present study, we were interested to investigate the effects of the social context (i.e. the presence of another conspecific producing request gesture and varying gaze direction) on the kinematics of action sequences performed by an individual. The sequence did not imply any relation with the conspecific.

In a previous kinematic study, Ferri et al. [11] observed that in a sequence in which the final intention was feeding a conspecific, the interaction of the giver with the receiver changed the kinematics of the movements if compared with the same sequence directed to an inanimate stimulus. Ferri et al. [11] proposed that the interaction is characterized by a social affordance that the giver activates on the basis of a social request produced by the receiver (the mouth aperture). The gaze of the receiver was a prerequisite to make a social request effective since a blindfolded receiver did not activate any social affordance. In the Ferri et al.'s study [11], the effects of approaching relations were only analyzed, that is the authors did not search for effects of request gestures aimed at executing movements whose direction and aim were different from those required by the task. This issue was addressed in the present study in which the participants were required to reach-grasp a bottle filled of orange juice and to lift it. The sequence was executed in presence of a conspecific who held or not an empty glass with his/her right hand. If the agent unconsciously interpreted the gesture of holding the glass as a pouring request, a corresponding motor program could be automatically activated and it could interfere with the different program required by the task.

In the Ferri et al.'s study [11], the availability of the gaze was tested: that is the effectiveness of the request gesture produced by a blindfolded receiver was compared with that of a not-blindfolded receiver. The gaze direction is also a stronger index of the intention of initiating a relation: indeed, the direct or averted gaze expresses an intention to interact or not, respectively [2], [3], [6]. In contrast, the gaze availability as compared to the not availability expresses the possibility to require an interaction. Consequently, the gaze availability is a condition necessary but not sufficient for requiring an interaction.

Sartori et al. [12] found results similar to those found by Ferri et al. [11]. These authors analyzed the effects of a request gesture of a conspecific (give-me-in-the-hand) on the kinematics of a sequence during which the agent reached-grasped an object and placed it in a container. However, these authors studied the effect of a sudden presentation of a request gesture on the control of movement execution; in contrast, we were interested in the effects of request gesture on planning the movement and consequently the request gesture was presented before movement onset. By the way, it has been proposed that different visual representations are used during planning and controlling the execution of a movement [13]. In addition, the gesture they presented (give-me-in-the-hand) implied touching the requiring conspecific. In contrast, we were interested in avoiding a potential direct contact with the conspecific, because the direct contact with a conspecific could increase the movement accuracy requirement. To this purpose, in the present study the final target of the request was a glass held in the actor's hand, and placed on the table.

We conducted four experiments. Experiment 1 was a baseline control experiment aimed at determining whether the presence of a conspecific not producing any request with a hand and gaze modified the kinematics of a sequence constituted by reaching-grasping a bottle filled of orange juice and lifting it. Experiments 2 and 3 aimed at determining whether the presence of a conspecific producing only a request of pouring with his/her hand, or only a request of interaction using his/her gaze, affected the sequence. Experiment 4 studied the effects of both hand gesture and eye contact.

## Experiment 1

**Figure 1:**
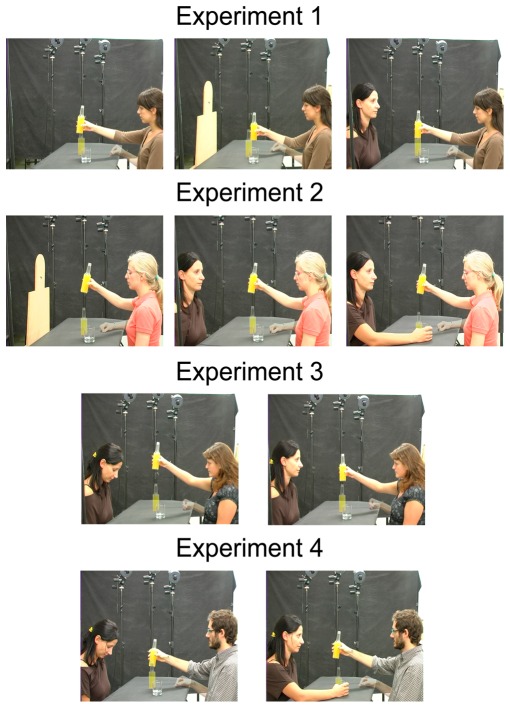
Experimental set-up and stimuli presented in experiments 1–4. Examples of the movements executed by the participants in the four experiments are shown. The actor and participants have seen the manuscript and figures and have provided written consent for publication.

Participants reached-grasped and lifted a bottle filled of orange juice in presence of an empty glass. We verified whether the presence of a conspecific (an experimenter) assuming a neutral posture (i.e. she was not producing any request) influenced the sequence (see Fig. 1). The conspecific presence was compared with the presence of a body-shape and with the absence of any stimuli in the scene (except the empty glass).

### Materials and methods

#### Participants

Eight naïve volunteers (3 females and 5 males, age 22–30 yrs.) took part in the experiment. All participants were right-handed [14] and without any history of neurological disorder or impairment. They were paid for their participation. The Ethics Committee of the Medical Faculty at the University of Parma approved the study. The experiments were conducted according to the principles expressed in the Declaration of Helsinki. All the participants in the present study provided written informed consent.

#### Apparatus and stimuli

The participants sat comfortably in front of a table on which they placed their right hand with the thumb and index finger in pinch position (Starting Position, SP). SP was along the participants' mid-sagittal plane and was 27 cm distant from their chest. A bottle (∼22 cm height, ∼5 cm diameter) filled with orange juice and an empty glass (∼8 cm eight, ∼7 cm width) were presented to the participants (Fig. 1). The bottle was placed on the table along the participants' mid-sagittal plane, 19 cm distant from SP, whereas the glass was placed on the left of the participant, 15 cm distant from bottle.

The bottle and the glass were presented alone or in presence of a conspecific (i.e. an experimenter), sitting in front of the participant, or a human body-shape, placed in front of the participants in the same position as the conspecific. The conspecific sitting in front of the participant placed her hands under the table and looked at a remote position beyond the participant's left cheek (neutral posture). The human body-shape was a wooden panel, the outline of which resembled the head and the upper trunk of a human body. The distance of the conspecific and the human body-shape from the participant was approximately 80 cm.

In 25% out of the trials, the glass was substituted by another bottle filled of orange juice presented in the same position of the glass.

#### Procedure

The participants performed a go-no-go task. In the go condition the empty glass was presented. The participants were required to reach, grasp and lift the bottle (Fig. 1). After each trial, the participant re-positioned the bottle on the initial target location. When the second bottle was presented on the table, the participants had to stay still and to wait for the next trial (no-go condition).

Each trial started with participant's eyes closed. When the experimenter gave the “GO” signal, the participant opened his/her eyes. They were required to look at the presented stimuli (target and scene) and, in the go condition, to reach, grasp and lift the bottle. No instruction was given about the height of the lifting movement. The participants grasped the bottle with their whole right hand (whole-hand-grasp, Fig. 1). Before the experiment onset the participants performed a training block of ten trials. The task was executed in a single block of 40 trials (10 trials/condition) and the order of stimulus presentation was semi-randomized. During the go condition, the participants were free to look at the scene as during natural interactions with objects and they were asked to perform the movement as naturally as possible.

#### Data recording

The movements of the participants' right arm were recorded using the 3D-optoelectronic SMART system (BTS Bioengineering, Milano, Italy). This system consists of six video cameras detecting infrared reflecting markers (spheres of 5-mm diameter) at a sampling rate of 120 Hz. Spatial resolution of the system is 0.3 mm. The infrared reflective markers were attached to the nail of the participant's right thumb and index finger; another marker was attached to the participant's right wrist. The markers on the thumb and index finger were used to analyse the grasp time course, whereas the wrist marker was used to analyse the time courses of reaching and lifting. The data of movement were analyzed with a software developed using MATLAB version 7.7 (R2008b). Recorded data were filtered using a Gaussian low pass smoothing filter (sigma value: 0.93). The grasp was studied by analyzing the time course of the distance between markers on the index finger and thumb. From a pinch position, the grasp is constituted by an initial phase of finger opening up to a maximum (maximal finger aperture) followed by a phase of finger closing on the object [15].

The time course of reach-grasp and lift was visually inspected: the beginning of the grasp was considered to be the first frame in which the distance between the two markers placed on the right finger tips increased more than 0.3 mm (spatial resolution of the system) with respect to the previous frame. The end of the grasp was the first frame after the beginning of finger closing in which the distance between the two right fingers decreased less than 0.3 mm with respect to the previous frame. The beginning of the reach was considered the first frame during which the displacement of the reach marker along any Cartesian body axis exceeded the value of 0.3 mm. To determine the end of the reach, we calculated the first frame following movement onset in which the X, Y and Z displacements of the reach marker were all less than 0.3 mm. Then, the frame temporally closer to the grasp end frame was chosen as the end of the reach. The frame immediately successive to the reach end was considered as the lift beginning, while the lift end corresponded to the frame in which the highest point of the hand trajectory was reached.

Concerning the grasp and reach parameters, we measured peak velocity of finger opening, reach peak velocity and reach peak elevation, i.e. maximal height of reach trajectory. Regarding the lift, we measured lift peak velocity, maximal curvature of participants' lift trajectory along the x and z axis. The maximal curvature is defined as the maximal distance (on x and z axis) of the wrist trajectory from the straight line connecting lift beginning and end. We analyzed peak velocities of the three components of the sequence in order to check whether the possible actions activated by the presence of the conspecific also affected the movement initial part, more related to planning. Curvatures in the trajectories were analyzed to search deviations due to inhibition effects of the movement elicited by the presence of conspecific or inanimate stimulus [16].

#### Data analysis

Repeated measures ANOVAs were carried out on the mean values of the grasping- reaching-lifting parameters. The within-subjects factor was scene stimuli (no stimulus vs human body-shape vs neutral posture conspecific). In all analyses post-hoc comparisons were performed using the Newman-Keuls procedure. The significance level was fixed at p = 0.05. When the factor was significant, we also calculated the effect size [η^2^_p(artial)_].

### Results and discussion

**Figure 2:**
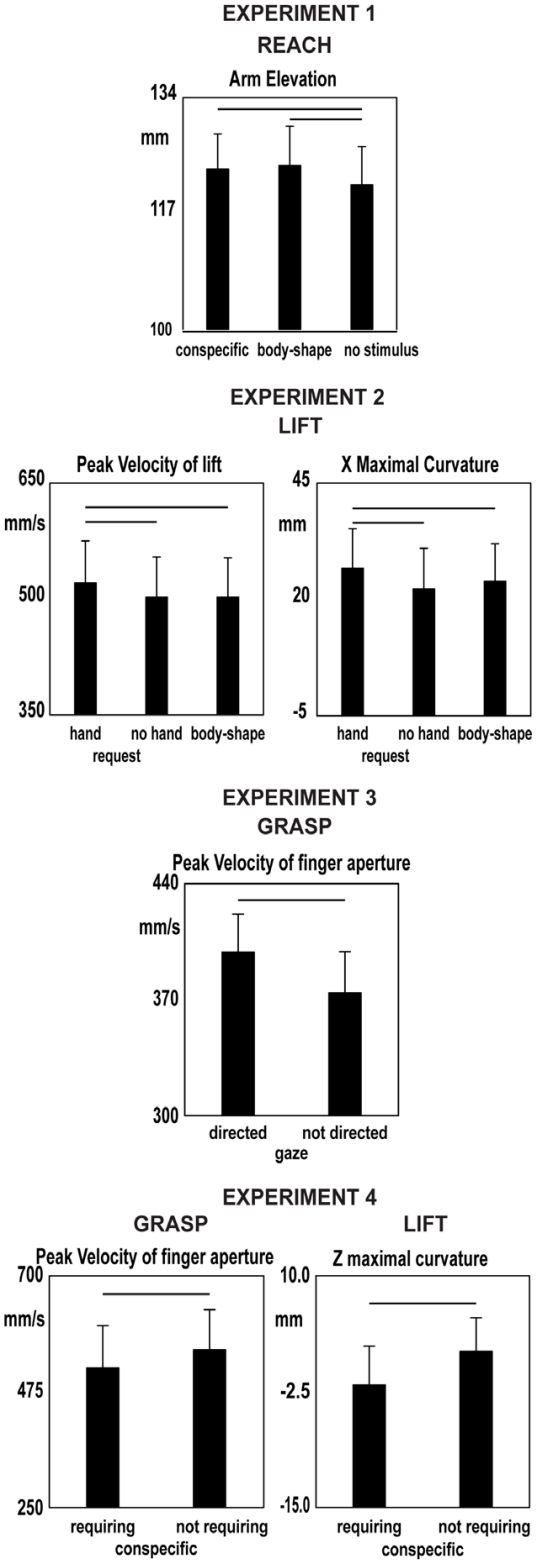
Kinematic parameters of grasp, reach and lift collected in experiments 1–4, which resulted significant in the ANOVAs. Vertical bars are SE, whereas horizontal bars indicate significance in the ANOVA. In the panel showing Z curvature, positive and negative values refer to movements directed to the left and to the right, respectively, whereas in the panel showing X maximal curvature positive values refer to backward directed movements.

Reach peak elevation was greater in the conditions of neutral posture of the conspecific (post-hoc test, p<0.05) and human body-shape (post-hoc test, p<0.05) as compared to no stimulus condition (F(2,14) = 4.0, p<0.05, η^2^_p_ = 0.4, Fig. 2). No difference (post-hoc test, p>0.05) was found between conspecific neutral posture and human body-shape. No other significance was found (Table 1)

**Table 1:** Results of statistical analyses performed on kinematic parameters.

	GRASP		REACH		LIFT	
	*Peak velocity of finger opening*	*Arm peak velocity*	*Arm elevation*	*Lift peak velocity*	*Z maximal curvature*	*X maximal curvature*
**Experiment 1**	F(2.7) = 0.82	F(2.7) = 0.13	F(2.7) = 3.97	F(2.7) = 0.72	F(2.7) = 0.46	F(2.7) = 0.63
Factor scene stimulus:	p>0.05	p>0.05	p<0.05	p>0.05	p>0.05	p>0.05
no stimulus vs human body shape vs neutral posture conspecific	n.s.	n.s.	η^2^_p_ = 0.4	n.s.	n.s.	n.s.
**Experiment 2**	F(2.7) = 0.1	F(2.7) = 0.08	F(2.7) = 1.99	F(2.7) = 3.69	F(2.7) = 1.71	F(2.7) = 4.06
Factor scene stimulus:	p>0.05	p>0.05	p>0.05	p<0.05	p>0.05	p<0.05
human body shape vs no hand request conspecific vs hand request conspecific	n.s.	n.s.	n.s.	η^2^_p_ = 0.4	n.s	η^2^_p_ = 0.4
**Experiment 3**	F(1.7) = 15.7	F(2.7) = 0.6	F(1.7) = 0.03	F(1.7) = 0.06	F(1.7) = 0.22	F(1.7) = 3.37
Factor gaze direction:	p = 0.005	p>0.05	p>0.05	p>0.05	p>0.05	p>0.05
directed vs not directed to the agent	η^2^_p_ = 0.7	n.s.	n.s.	n.s.	n.s.	n.s.
**Experiment 4**	F(1.7) = 9.36	F(1.7) = 0.99	F(1.7) = 0.00	F(1.7) = 0.02	F(1.7) = 7.42	F(1.7) = 0.55
Factor conspecific's posture:	p<0.05	p>0.05	p>0.05	p>0.05	p<0.05	p>0.05
not requiring vs requiring	η^2^_p_ = 0.6	n.s.	n.s.	n.s.	η^2^_p_ = 0.5	n.s.

The presentation of a conspecific assuming a neutral posture (i.e. the experimenter did not produce any hand request gesture and did not look at the participant) did not establish a social interaction between the participant and the conspecific. In fact, even if the reach trajectory was higher in the condition of conspecific presentation compared with the absence of any stimulus, the same effect was observed when the conspecific was substituted by the human body-shape. Consequently, the social context was not responsible for the modification observed in the trajectory. Probably, higher trajectories were due to a different control of movement because, in addition to egocentric references, the position of the conspecific or the human body-shape could be used as allocentric reference, that is spatial reference with respect to the target and the arm position [17].

## Experiment 2

We wanted to asses whether the only conspecific's gesture of holding the glass with the hand affected the reach, grasp and lift components of the sequence executed by the participant. This could suggest that a social request of pouring could be activated.

### Materials and methods

#### Participants

A new sample of eight right-handed [14] naïve volunteers (4 females and 4 males, age 20–29 yrs) took part in the experiment.

#### Apparatus, stimuli and procedure

Apparatus and procedure were the same as in experiment 1. The bottle and the empty glass were always placed on the table in presence of the human body-shape or the conspecific who could assume two different postures: a not-requiring-with-the-hand posture (invisible hands placed under the table) or a requiring-with-hand posture (right hand holding the empty glass). In all conditions the conspecific's gaze was directed beyond the participant's left cheek. (Fig. 1). The no-stimulus condition was not included in the procedure; consequently the experimental session consisted of a single block of 40 trials.

#### Data recording and analysis

Data recording was the same as in experiment 1. Repeated measures ANOVAs were carried out on the mean values of the reaching, grasping, and lifting parameters. The within-subjects factor was scene stimuli (human body-shape vs no hand request conspecific vs hand request conspecific). In all analyses post-hoc comparisons were performed using the Newman-Keuls procedure. The significance level was fixed at p = 0.05. For every significant factor, we also calculated the effect size [η^2^_p(artial)_].

### Results and discussion

The lift component was affected by the request gesture (presence of the conspecific holding the glass with her hand). Peak of velocity of lift increased (F(2,14) = 3.9, p = 0.05, η^2^_p_ = 0.4, Fig. 2); post-hoc analysis (p<0.05) showed that in the condition of request gesture the participants lifted the bottle faster than in the other experimental conditions which did not differ from each other (p>0.05). The maximal x curvature was also affected by the request gesture. The lift trajectory showed a back deviation (F(2,14) = 4.1, p<0.05, η^2^_p_ = 0.4, Fig. 2). Post-hoc analysis (p<0.05) showed a significant difference between this condition and the other two. No significant effect was found between not-requiring-with-the-hand conspecific and the human body-shape conditions (p>0.05). No other significance was found (Table 1)

It may be that when the conspecific held the empty glass with her hand, she produced a request gesture of pouring. Thus, it is possible that the participant's lift movement was faster and directed back in order to avoid satisfying that request. In other words, the pouring program could interfere with the sequence inducing a deviation away from the glass. In fact, the participant was required to lift rather than to pour. Another possibility to explain the different kinematics could be that the participant automatically tended to avoid the conspecific's forearm and hand which were at reaching distance (i.e. in the participant's peripersonal space). However, if the hand and forearm were simple distractors, the trajectory of also approaching the target should be deviated away from the distractor and/or movement should be slowed down as previously found [16], [18]–[23] (see also De Stefani, Innocenti, Campione, Bernardi, Gentilucci, in press). This was not found. Summing up, the hand request gesture was effective in the last movement of lifting when, in the case of actual pouring, the trajectory deviates towards the glass. The fact that no effect was initially found, especially during grasping, could be due to weaker conspecific's request.

In experiments 1–2, the conspecific's gaze was directed beyond the participant rather towards the participant. If someone looks at a conspecific, it is possible that this occurs because he/she is interested in him/her and wishes to establish a relation [2], [3], [6]. On the contrary, if he/she looks away, he/she is not interested. In this sense, the presence of the hand on the glass may be considered an incomplete request when the conspecific looked at a remote position. Indeed, the participant could be initially doubtful if the conspecific really wanted to interact with him/her if no eye contact was established. Consequently, we tested whether eye contact played a role in modifying sequences performed in a social context.

## Experiment 3

Participants reached-grasped and lifted a bottle in presence of an empty glass. In the scene, a conspecific (an experimenter) was present. She looked at the participant (direct gaze condition) or directed her gaze down (not directed gaze condition). No request gesture was produced with the hands (the conspecific's hands were placed under the table).

### Materials and methods

#### Participants

A new sample of eight right-handed [14], naïve volunteers (4 females and 4 males, age 20–29 yrs) took part in the experiment.

#### Apparatus, stimuli and procedure

Apparatus and procedure were the same as in experiment 1. The conspecific presented in the scene looked at two different locations: towards the participant's eyes or down. The conspecific hands were placed under the table (Fig. 1). The experimental session consisted of a single block of 30 trials.

#### Data recording and analysis

Data recording was the same as in experiment 1. Repeated measures ANOVAs were carried out on the mean values of the reaching-grasping-lifting parameters. The within-subjects factor was conspecific's gaze direction (directed vs not directed to the agent). The significance level was fixed at p = 0.05. When the factor was significant, we also calculated the effect size [η^2^_p(artial)_].

### Results and discussion

Only the grasp component was affected by the cospecific's gaze direction. The participants opened their fingers faster in the direct gaze condition (peak velocity of finger opening; F(1,7) = 15.7, p = 0.005, η^2^_p_ = 0.7, Fig. 2). No other significance was found (Table 1)

The finding that fingers were opened more quickly when the conspecific's gaze was directed towards the agent probably depended on the fact that the direct gaze could be interpreted as a performance request, i.e. as a request to execute quickly the actually required task (i.e. to reach-grasp and to lift the bottle), since no request gesture concerning the interactions between glass and bottle was produced with the actor's hand.

The sole request gesture of holding the glass with the hand influenced the last action of lifting (experiment 2), i.e. when the trajectory of a possible pouring deviated from that of lifting. Moreover, the sole gaze directed towards the agent induced an effect of urgency on grasping execution (experiment 3), i.e. it produced a request on the initial phase of the sequence. Thus, the interaction between gaze and request gesture could influence the entire sequence.

## Experiment 4

Participants reached-grasped and lifted a bottle filled of orange juice in presence of both an empty glass and a conspecific. The conspecific could assume a not requiring attitude or a requiring attitude.

### Materials and methods

#### Participants

A new sample of eight right-handed [14], naïve volunteers (5 females and 3 males, age 20–29 yrs) took part in the experiment.

#### Apparatus, stimuli and procedure

Apparatus and procedure were the same as in experiment 1. The conspecific (an experimenter) facing the agent could assume a not requiring attitude (hands placed under the table and gaze directed down) or a requiring attitude (right hand holding the empty glass and gaze directed towards participant's eyes). (Fig. 1). The experimental session consisted of a single block of 30 trials.

#### Data recording and analysis

Data recording was the same as in experiment 1. Repeated measures ANOVAs were carried out on the mean values of the grasping-reaching-lifting parameters. The within-subjects factor was conspecific's attitude (not requiring vs requiring). The significance level was fixed at p = 0.05. When the factor was significant, we also calculated the effect size [η^2^_p(artial)_].

### Results and discussion

The participants opened their fingers more slowly in the condition of conspecific's requiring attitude (F(1,7) = 9.4, p<0.05, η^2^_p_ = 0.6, Fig. 2). The maximal z curvature of lift was affected by the conspecific's attitude (F(1,7) = 7.4, p<0.05, η^2^_p_ = 0.5, Fig. 2). The participant's lift trajectory veered away from the glass (i.e. it was directed to the right) when the conspecific produced a request with both her hand and eyes. No other significance was found (Table 1).

The grasp and lift were interfered by the presence of a conspecific who produced the request gesture of pouring and looked towards the agent. It is likely that an automatically activated program of pouring interfered with the required sequence inducing a slowing down of the finger opening and a deviation of the lift trajectory away from the requiring conspecific. In fact, the participant was required to lift the bottle rather than to pour the liquid of the bottle into the glass. Sartori et al. [12] found that request gesture induced a deviation of arm trajectory towards the hand of the requiring conspecific. This result contrasting with those of experiments 2 and 4 can be explained by the fact that these authors presented a sudden request during movement execution, whereas we presented a request before movement initiation. Consequently, in the first case the request might have produced an assimilative effect, whereas in the second case it might have produced a contrastive effect. This might have been consequent to the time at disposal for elaboration of a response.

## Discussion

The human actions occur individually or within a social interaction: in the latter case the human actions depend on the presence of other persons and their relevant intentions. For example, such a causal dependence can be found in the action of reach to grasp and lift a bottle. We could execute this movement because we would like to pour some liquid into a glass and, obviously, we can do it for ourselves. Nevertheless, the same action can take place in interactions, taking into account at least another person as part of one's reason for acting. In everyday life, the other person's body posture may provide an important cue in discriminating whether two agents are acting for a shared goal or individual purposes [24]. For an example, a conspecific holding an empty glass in his/her hand can express the request of pouring the liquid of the bottle into the glass in order to drink. Moreover, gaze direction is a potent social cue, which is indicative of other persons' intentions [6]. During social interactions, people's eyes convey a wealth of information about the direction of their attention and their emotional and mental state [25]. The main objective of the present study was to test for a relation between the social context in which an action takes place and the availability of actors' hand posture and gaze (pertinence of a request gesture).

In experiment 1, a conspecific (the potential receiver) assumed a neutral attitude with her hand and eyes. The results showed that the agent (the potential giver) did not establish a social interaction with the conspecific. In fact, the conspecific presence produced the same effects as a human body-shape when the two stimuli were compared with the absence of any stimulus in the scene.

In experiment 2, the sequence was affected by the presence of the conspecific producing the request gesture of pouring (holding the glass with the hand). Specifically, the pouring request interfered with the execution of the final lifting, i.e. when the trajectory could deviate from the potential trajectory of pouring requested by the hand gesture. The finding that no initial effect (i.e. on reaching-grasping) due to the hand gesture was found could depend on an incomplete request. In fact, the gaze was directed beyond the agent. It is well known [2], [3], [6] that the gaze directed to a conspecific is an index of the intention to interact with him/her, whereas an averted gaze expresses the intention of non interaction. Consequently, a direct gaze could make effective the hand request of pouring.

In experiment 3, we tested the effects of the sole conspecific's gaze (direct vs not-directed to the agent) without production of pouring request gesture with the hand. The results showed a quicker grasp in direct gaze condition. Observing another's gaze direction affects arousal and can modulate judgments about the observed scene [26]. Indeed, knowing, for example, whether you are the target of another person's gaze because you are a possible adversary or partner is valuable information as it facilitates the generation of a contextually appropriate behavioural response. Direct gaze has also been shown to activate neural circuits that are associated with the appraisal (e.g., threat, reward value) of social stimuli. Imaging investigations have demonstrated increased activity in the superior temporal sulcus, amygdala, and ventral striatum when people view faces displaying direct gaze [7], [27], [28]. It is possible that, in the present study, the direct gaze of the conspecific was interpreted either as a quick performance request since no hand request was produced or it induced embarrass in the participant. Indeed some times fixating a conspecific does not necessarily imply a successive interaction with a conspecific. It can express a command, to execute quickly the previously assigned task (lifting the bottle), and fixating the conspecific continues until the task is not accomplished. An alternative explanation of this finding is that direct gaze can also signal interests and precedes social interaction. It is thus possible that participants expected the conspecifics to start an interaction, and to avoid interference with the assigned task (lifting the bottle), speeded up the grasping of the object.

In experiment 4, the agents were interfered by the condition of conspecific's requiring hand posture and direct gaze. In other words, the direct gaze made effective the hand request even during grasping. Probably, a program of pouring initially activated by the gaze interfered by slowing down the grasp. Concerning the lift, a deviation away from the glass was induced.

The results of the present study are in agreement with those by Ferri et al.'s study [11]. These authors found that the conspecific's request to be fed (opened mouth) induced a slowing down of the sequence of reaching-grasping and feeding. This effect disappeared when the conspecific was blindfolded. That is, the availability of the gaze of the requiring conspecific was prerequisite to make effective the request. However, in the Ferri et al.'s study [11] the conspecific looked at a remote position beyond the agent. This type of gaze was not effective for the initial reaching-grasping in the present study where, on the contrary, fixating the conspecific was necessary in order to affect the grasp. It is possible that the conspecific's request of being fed, i.e. the mouth aperture, was enough strong to need of even weaker gaze request to be effective as compared to the request of pouring. Indeed, the request to be fed was unequivocal since a movement of approaching the receiver was required. In contrast, the request expressed by the holding of the glass could induce uncertainty in the gesture interpretation since the task required a movement different from that required by the gesture. Consequently, initially the posture of the actor could be also interpreted, for example, as the actor's intention of placing the glass in a different location. This could be avoided if the direct gaze was used to provide an initial request of direct interaction with the agent.

The results of the present study suggest that in a social context (i.e. in presence of conspecific(s)) mechanisms of complementary actions can be activated. Indeed, the presence of a conspecific requiring pouring, automatically activated in the agent that pouring program that in turn influenced the actual action. This may be considered at odds with the idea about a mirror circuit automatically activated by gesture observation in order to understand the meaning of the action [29]. However, we can speculate that a program complementary to the request can be activated as soon as understanding the meaning of the observed request [30]. Consequently, the two processes can be strictly related. This is supported by the data by Newman-Norlund et al. [31] who found that preparation of simulation and complementary actions activated the same frontal and parietal circuit. Activation for complementary actions was greater than for simulation ones. Greater activation suggests primary function of area,

In a previous study, De Stefani et al. (in press) found that when reaching-grasping and lifting a bottle filled of orange juice, the presence of an empty glass affected the kinematics of the sequence when compared to the presence of a filled glass. The authors interpreted this result as due to automatic activation of a pouring program which interfered with the actual sequence. The results of the present study, in which an empty glass was always presented, adds the notion that the manual request gesture of pouring produced by a conspecific and her gaze induced a further effect on the kinematics of the actual sequence. This occurred because joint actions were activated [32]–[33]. In sum, the presentation of an empty glass activated an actually functional (“working”, De Stefani et al. in press) affordance, i.e. a motor program representing the interaction between two functionally compatible artefacts. In contrast, the presentation of a conspecific producing a request gesture activated a social affordance whose kinematic effects on the sequence were different from and additive to those of the working affordance. In fact, the social affordance also takes into account the type of interaction between conspecifics in addition to the function of the object (see for an example the kinematic modification during joint actions of cooperation and competition [34]). In other words, the working affordance codes “what” to do with the object regardless of the social context, whereas the social affordance might code “how” to interact with the object when this can be also shared with conspecific(s) in relation to a social context.

The results of the present study can generate interesting predictions in the case of Autism disorder (AD). AD is a heterogeneous developmental syndrome characterized by a marked impairment in social interaction and communication [35]. We propose that AD patients can be impaired in recognizing and responding to hand gestures requiring execution of motor sequences guided by a specific intention. The deficit can also concern the effects of eye contact on request gesture, since gaze direction of conspecifics causes in AD patients behaviour different from that of healthy individuals [36]–[38]. The application of the paradigm of the present study to AD patients can determine whether, unlike healthy individuals, the kinematics of their motor sequences are not affected by conspecifics' hand request gesture and/or gaze direction. In other words, autism syndrome can produce inability to understand the intentions of conspecifics by observing their gestures and, consequently, to produce interactions like complementary actions.
