# Correction: Gaze Direction and Request Gesture in Social Interactions

**DOI:** 10.1371/journal.pone.0091377

**Published:** 2014-02-27

**Authors:** The PLOS ONE Staff

An affiliation for the first author is incorrectly omitted. In addition to institution number 1, Alessandro Innocenti is also affiliated with the following institution: Health Sciences Department, Milan University, Milano, Italy.
